# A real‐life snapshot: Evaluating exposures to low energy availability in male athletes from various sports

**DOI:** 10.14814/phy2.16112

**Published:** 2024-06-23

**Authors:** Birna Vardardottir, Anna S. Olafsdottir, Sigridur Lara Gudmundsdottir

**Affiliations:** ^1^ Faculty of Health Promotion, Sport & Leisure Studies University of Iceland Reykjavik Iceland

**Keywords:** energy availability, male athletes, nutrition status, relative energy deficiency, sport nutrition

## Abstract

Problematic low energy availability (LEA) is the underlying cause of relative energy deficiency in sport (REDs). Male specific etiology, as well as the duration and degree of LEA exposures resulting in REDs remain to be adequately described. The present study aimed to assess occurrences of LEA (energy availability [EA] <25 kcal/kg fat‐free mass/day) in male athletes from various sports over 7 days. Associations between number of LEA days, physiological measures, and body image concerns were subsequently evaluated. The athletes recorded their weighed food intakes and training via photo‐assisted mobile application. Body composition and resting metabolic rates were measured, and venous blood samples collected for assessments of hormonal and nutrition status. Participants also answered the Low Energy Availability in Males Questionnaire (LEAM‐Q), Eating Disorder Examination—Questionnaire Short (EDE‐QS), Exercise Addiction Inventory (EAI), and Muscle Dysmorphic Disorder Inventory (MDDI). Of 19 participants, 13 had 0–2, 6 had 3–5, and none had 6–7 LEA days. No associations were found between the number of LEA days with the physiological and body image outcomes, although those with greatest number of LEA days had highest EEE but relatively low dietary intakes. In conclusion, this group displayed considerable day‐to‐day EA fluctuations but no indication of problematic LEA.

## 1 INTRODUCTION

The vast majority of sport science research has been conducted on males, with the explicit exception of relative energy deficiency in sport (REDs). Accordingly, only 20% of athletes featured in original RED investigations between 2018 and 2023 were males (Mountjoy et al., 2023). This has left a gap in the scientific understanding on male‐specific etiology and consequences of REDs (Hackney et al., 2023). Problematic (i.e., severe or prolonged) low energy availability (LEA) is the underlying culprit of REDs, but the duration and degree of LEA resulting in a problematic scenario is yet to be defined (Burke et al., 2023). The often‐used energy availability (EA) cutoff of 30 kcal/kg fat‐free mass (FFM) comes from laboratory‐based studies on nonathletic females in early 2000s, where menstrual disturbances occurred at EA levels below this threshold (Ihle & Loucks, 2004; Loucks & Thuma, 2003). The universal applicability of such a cutoff has been questioned due to individual and sex differences (De Souza et al., 2019; Mountjoy et al., 2023). It has further been suggested that if a male‐specific threshold would exist it would be lower than for females, or somewhere in the range of 9–25 kcal/kg FFM/day (Jurov et al., 2021; Jurov, Keay, & Rauter, 2022; Lane et al., 2021; Sim et al., 2024), with male bodies proposedly more physiologically resistant to energy deficiency (Mountjoy et al., 2023).

Among factors that confuse establishment of reliable LEA cutoffs is that EA and its components, energy intake and exercise energy expenditure (EEE), tend to fluctuate markedly from day‐to‐day in real life (Taylor et al., 2022). In accordance, adaptable LEA refers to an occasional or short‐term exposures to LEA and can as part of periodized nutrition protocols stimulate training adaptations (Mountjoy et al., 2023; Mujika et al., 2018). There may also be circumstances where weight or body composition manipulations, and thus slight or temporary reductions in EA, are reasonably needed. Weight or body composition goals must, however, always be approached in a way that does not sacrifice athletes' mental and/or physical health (Mathisen et al., 2023).

Low or reduced testosterone is a primary male‐specific indicator of REDs, as defined by the International Olympic Committee (IOC), while other proposed physiological perturbations are supported by weaker evidence (Hackney et al., 2023; Mountjoy et al., 2023; Stellingwerff et al., 2023). The Low Energy Availability in Males Questionnaire (LEAM‐Q) (Lundy et al., 2022) was recently developed and resembles an older screening tool for physiological complications of LEA in females (LEAF‐Q) (Melin et al., 2014). However, validated LEAM‐Q scores and cutoffs, other than for sex drive, could not be derived due to lack of associations between the generated scores and clinical markers (Lundy et al., 2022). In addition, disordered eating (DE) and eating disorders (ED) are well known risk factors of REDs (Torstveit et al., 2019; Vardardottir et al., 2023) but most available screening tools for ED and DE are primarily focused on female specific symptoms and/or concerns. Therefore, it has been suggested that questionnaires or items used to screen for muscle dysmorphic traits could be better for capturing the problem in males and sports where muscularity is perceived as advantageous (Forrest et al., 2019; Prnjak et al., 2022). Consequently, screening for muscle dysmorphia could play a role in early detection of REDs. Some symptoms are shared between DE/ED and muscle dysmorphia, including fear of gaining body fat or strong desire to become leaner (Vardardottir et al., 2023). REDs may also occur unwittingly when athletes are exposed to problematic LEA due to unawareness of their energy requirements or very high EEE that make sufficient fuelling a challenge (Melin et al., 2023).

The primary ambition of dedicated sport practitioners and scientists alike is to help athletes maximize their potentials, without causing harmful health effects (Hackney et al., 2023). Therefore, it is important to understand how much energy‐related stress an athlete can tolerate and adapt to, and when it becomes too much. The present study aimed to assess occurrences of LEA exposures in males from various sports over 7 days. Associations of the number of LEA days with dietary intake, physiological measures, and body image concerns were subsequently evaluated.

## 2 METHODS

### 2.1 Study population

This study used data from male participants in the RED‐I research project which aim was to evaluate EA and risk factors of REDs in high‐level Icelandic athletes. As precisely described elsewhere (Vardardottir et al., 2023), athletes had to be at least 15 years old and defined by the National Olympic Committee of Iceland as either elite or sub‐elite. The athletes came from six different sport groups that were defined according to published literature: ball, endurance, aesthetic, technical, weight‐class, and power sports (Torstveit & Sundgot‐Borgen, 2005; Vardardottir et al., 2023). The research was conducted in two parts: (1) Online questionnaire (July to December 2021) and (2) Two laboratory visits and assessments of dietary intake and EA over seven consecutive days (April to September 2022). All eligible respondents with complete or near‐complete response to the online questionnaire were invited to the second part. In the case of athletes under 18 years old the invitation was sent to both them and their parents/legal guardians. Those who did not respond to the initial invitation received at least two reminders. The study protocol was approved by the Icelandic Ethics Committee (VSNb2021050006/03.01). Informed written consent was collected from all participants prior to participation.

### 2.2 Low Energy Availability in Males Questionnaire

Participants responded to the LEAM‐Q and demographic questions online via the Qualtrics XM Platform in the first part of the study. LEAM‐Q was recently developed for screening of physiological symptoms of REDs in athletic males. LEAM‐Q consists of several categories: dizziness, gastrointestinal function, resting thermoregulation, injury and illness interfering with training and competition, fatigue (lethargy, tiredness, difficulties with concentration), fitness (body aches, stiff muscles, physical exhaustion, and injury proneness), sleep, recovery (physical recovery and perceived training progress), energy levels (training readiness, perceived happiness, and energetic levels), and sex drive (general sex drive rating and average number of weekly morning erections). Of the various LEAM‐Q outcomes, validated scores are only available for sex drive. A low sex drive is indicated if respondents (A) rate their sex drive as low or report that they do not have much interest in sex OR (B) report rarely or never having morning erections (in the past month) AND consider that to be a little or much less frequent than what is normal for themselves (Lundy et al., 2022). Responses to the other LEAM‐Q items and prevalence of negative/undesirable outcomes were also evaluated.

### 2.3 Laboratory measurements

Participants included in the second part of the study arrived at the first lab visit in a fasted state in the morning. Body composition was measured with whole body Dual Energy X‐Ray Absorptiometry (GE Lunar iDXA; GE Medical Systems, Belgium) and a venous blood sample was drawn for assessment of hormonal and nutrition status. The blood samples were analyzed for serum testosterone, thyroid stimulating hormone (TSH), Immunoglobulin A (IgA), iron (Fe), ferritin, total‐iron‐binding‐capacity (TIBC), 25‐OH‐Vitamin D (25(OH)D), vitamin B12, calcium, and magnesium. Testosterone, 25(OH)D, and vitamin B12 were measured on Roche Cobas® 801 (Electrochemiluminescence binding assay/competition principle). Fe and TIBC were measured on Cobas 702, with Fe measured via colorimetric assay (FerroZine method without deproteinization). Unsaturated IBC (UIBC) was assessed via direct determination with the FerroZine method and sum of serum Fe and UIBC represents the TIBC. Ferritin and TSH were measured on Cobas 801 (Electrochemiluminescence binding assay/Sandwich principle). IgA, calcium and magnesium were measured on Cobas 702. IgA was assessed via immunoturbidimetric assay and magnesium with colorimetric assay. The reaction of calcium ions with NM‐BAPTA forms a complex, which subsequently reacts with EDTA, and the absorbance change is directly proportional to the calcium concentration (measured photometrically). Testosterone levels <8 nmol/L were considered clinically low and 8–12 nmol/L as sub‐clinically low (Fredericson et al., 2021). 25(OH)D concentrations <30 nmol/L were regarded as deficient, <50 nmol/L as insufficient (Itkonen et al., 2021), and <80 nmol/L as below recommendations for athletes (Mountjoy et al., 2018; Tuma et al., 2023). Markers of insufficient iron levels were low Fe (<10 μmol/L), low ferritin (adolescents <23 μg/L, adults <30 μg/L), and high TIBC (>70 μmol/L). Transferrin saturation (TSAT) was calculated by dividing Fe with TIBC and multiply by 100. TSAT <20% has been used as an indication of iron deficiency (Reinke et al., 2012). The reference ranges used for TSH was 0.4–4.0 mU/L, IgA 0.7–3.7 g/L, vitamin B12 142–725 pmol/L, calcium 2.15–2.6 mmol/L, and magnesium 0.74–0.99 mmol/L, as defined by the laboratory. Approximately 2 weeks after the initial visit, participants arrived again in a fasted state in the morning at another lab for RMR measurements via indirect calorimetry (ventilated hood; Vyntus CPX). RMR was also estimated from the Cunningham formula (Cunningham, 1991) and RMR ratio was calculated by dividing measured with estimated RMR. RMR ratio <0.90 is among suggested markers of REDs (Stellingwerff et al., 2023; Sterringer & Larson‐Meyer, 2022). FFM index (FFMI) was calculated by dividing total FFM in kg with height in meters squared (Kyle et al., 2003). The DXA, blood analyses and RMR measurements have been described in further details elsewhere (Vardardottir et al., 2023).

### 2.4 Energy availability and dietary intake

#### 2.4.1 Digital food and training logs

At the end of the first lab visit participants were asked to install a mobile application (app), on their phones and received encoded login info. Thereafter they were verbally instructed on how to use the app for seven consecutive days. Detailed written instructions were also provided, and all were carefully informed about the aims of the dietary and training assessments. Participants were then instructed not to change their dietary and/or training behaviors due to participation in the study. The app was in Icelandic and sent daily reminders during the registration period. Further information about the app and the rationale behind remote food and training photography methods are provided in a previous female specific publication from the RED‐I study (Vardardottir et al., 2024).

**FIGURE 1:**
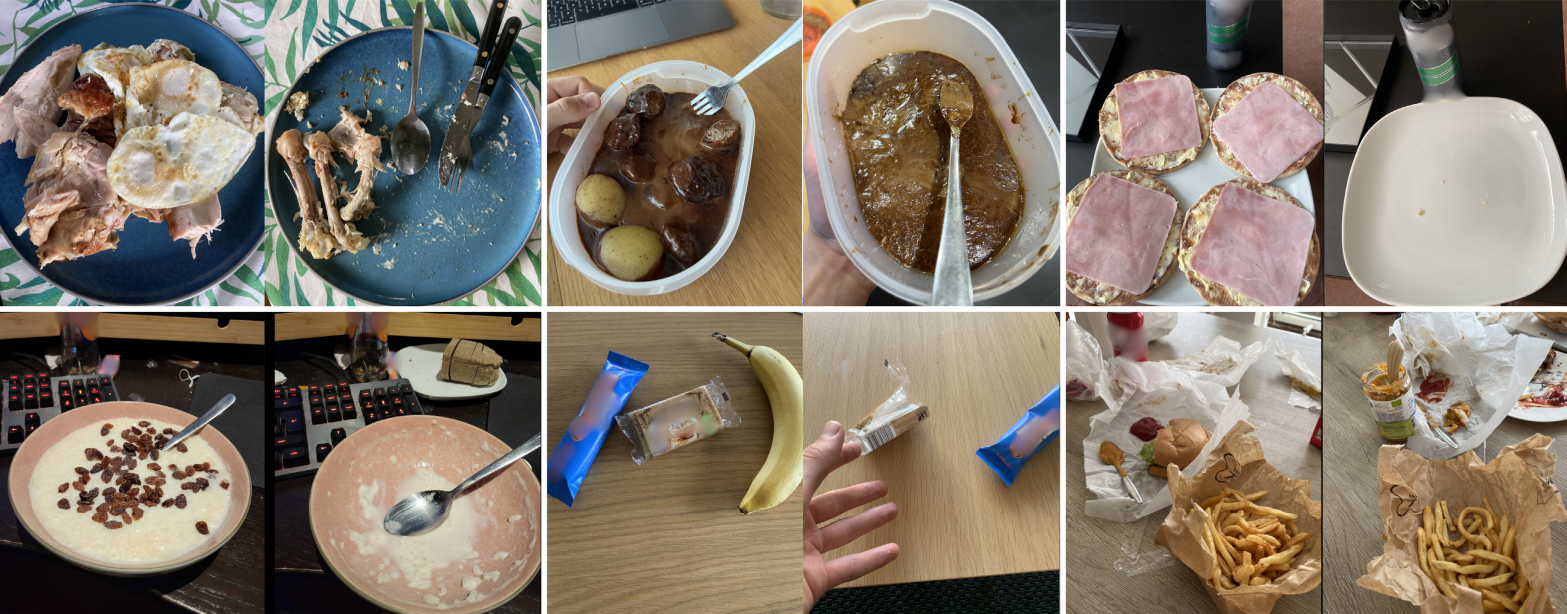
Examples of before and after photos from meals registered in the mobile application. The examples are random and from six different individuals.

Briefly, the athletes logged all foods, beverages other than still water, and supplements in the app. They were asked to weigh all foods (kitchen scales were provided if needed) and upload before and after photos of all meals and snacks. Examples of meal photos registered in the app are shown in Figure 1. During the same period, all completed training sessions were recorded in the app with information on type of training, duration, and intensity. Before starting the app registration, the athletes were asked about average number of weekly training hours via questionnaire. The food photography readings and interpretation were based on a validated approach (Olafsdottir et al., 2016). Researchers and assistants involved with screening, coding, and calculations from registered data all had separate tasks in the process which they were specially trained for.

#### 2.4.2 Dietary intake and energy availability calculations

After coding (i.e., assigning each meal item an identifying code for linkage with the Icelandic food composition database, ISGEM), food records data were transported to the ICEFOOD calculation program for calculations of energy, macro‐ and micronutrient intakes, as described earlier (Vardardottir et al., 2024). Estimations of EEE were based on registered training information; exercise mode, intensity and/or perceived exertion, and duration. Training sessions, or different sessions' parts, were assigned a metabolic equivalent (MET) value relevant for the activity type and intensity (Ainsworth et al., 2000). Total EEE was calculated by multiplying the MET scores with the session/activity duration. Measured or estimated RMR was then subtracted from total EEE to only capture the energy cost of the exercise.

Daily EA was calculated using the following formula (Loucks et al., 2011):

$$\text{Energy availability}=\frac{\text{Energy intake}\;\left(\text{kcal}\right)-\text{Exercise energy expenditure}\;\left(\text{kcal}\right)}{\mathrm{Fat}\;\text{free mass}\;\left(\mathrm{kg}\right)}$$

A threshold of 25 kcal/kg FFM/day was used to define LEA, as this is the upper level of the currently proposed LEA range (Jurov, Keay, & Rauter, 2022; Mountjoy et al., 2023). Accordingly, number of LEA days hereafter refer to the number of registered days below this value. When applicable, percentage with macro‐ and micronutrient intakes below reference values were checked. Low intake of carbohydrate intake was defined as mean intakes <4.0 g/kg/day (Burke et al., 2011; Slater & Phillips, 2013) and low protein intake <1.5 g/kg/day (Knuiman et al., 2018; Slater & Phillips, 2013). Low daily intakes of fiber (<25 g) (Carlsen & Pajari, 2023), vitamin D (<15 μg) (Itkonen et al., 2021), iron (<9 mg) (Domellöf & Sjöberg, 2024), folate (<300 μg) (Bjørke‐Monsen & Ueland, 2023), and vitamin B12 (<3.2 μg) (Bjørke‐Monsen & Lysne, 2023) were defined according to the Icelandic and Nordic nutrition recommendations.

### 2.5 Disordered eating, muscle dysmorphia, and compulsive exercise

As part of the initial lab visit, the athletes were asked to answer three brief questionnaires that screen for DE, muscle dysmorphia and compulsive exercise symptoms: Eating Disorder Examination—Questionnaire Short (EDE‐QS) (Gideon et al., 2016), Muscle Dysmorphic Disorder Inventory (MDDI) (Hildebrandt et al., 2004), and Exercise Addiction Inventory (EAI) (Terry et al., 2004). Each of the 12 EDE‐QS items are rated on a 4‐point scale (score 0–3), with the cutoff score set at ≥15. The 13 MDDI items are rated on a 5‐point scale (score 1–5), with the cut off score set at ≥39. EAI consists of six items rated on a 5‐point scale (score 1–5). Individuals scoring ≥24 on EAI are considered at risk of compulsive exercise, while 13–23 indicates some symptoms, and 6–12 no symptoms.

### 2.6 Data analysis

SPSS 29.0.1.1 and GraphPad Prism 10 were used for statistical analyses, with significance set to *α* < 0.05. Distribution of continuous variables was checked with Shapiro–Wilk and Q‐Q plot observations. Continuous variables were summarized as mean ± SD for normally distributed data, and medians with 25 and 75 interquartile (IQR) ranges for nonparametric data. When applicable, a comparison of intakes and serum status of certain nutrients between participants using supplements containing the nutrient of interest and those who did not was conducted using independent samples *t*‐test. Correlations of dietary intakes, physiological measures and scores on EDE‐QS, EAI and MDDI with number of LEA days were evaluated with the Pearson's r coefficient for parametric and Spearman's rank coefficient for nonparametric data. Simple linear regression was applied for graphical illustrations of the best‐fit line.

## 3 RESULTS

### 3.1 Participants and study flow

**FIGURE 2:**
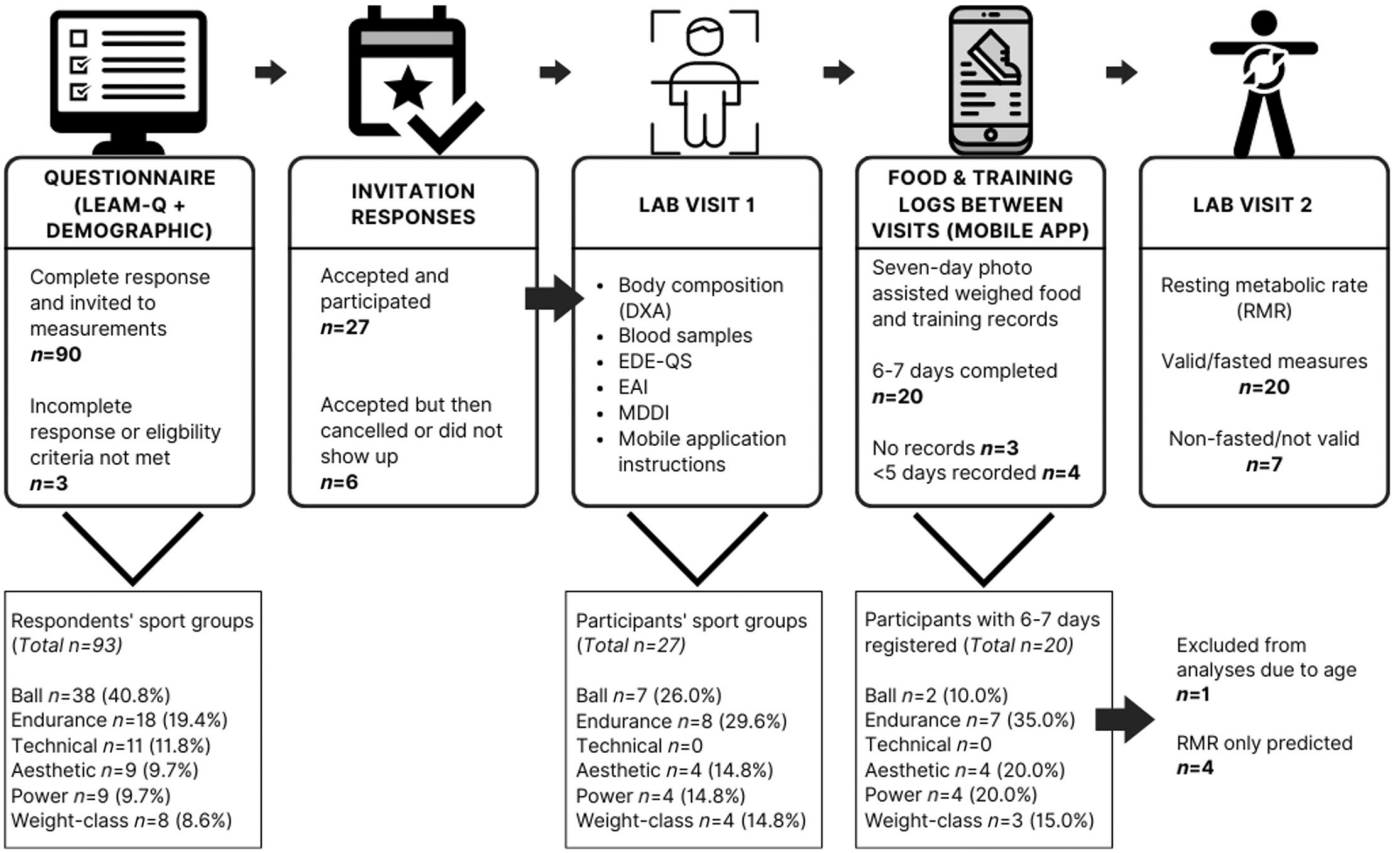
Flow of participants through the study. LEAM‐Q: Low Energy Availability in Males Questionnaire; DXA: dual energy x‐ray absorptiometry; EDE‐QS: Eating Disorder Examination–Questionnaire short; EAI: exercise addiction inventory; MDDI: muscle dysmorphic disorder inventory. Ball sports: football, handball, basketball, volleyball, table tennis, badminton; Endurance: middle to long distance running, swimming, cycling, triathlon; Technical: golf, horse riding, archery; Aesthetic: gymnastics, ballroom dancing, ballet; Power: sprinting, throwing, and jumping events, alpine skiing; Weight‐class: powerlifting, weightlifting, taekwondo, judo.

A total of 93 (23.5 ± 9.5 years) athletes responded to the online questionnaire. Thereof, three only completed a small part of it or did not fulfill the eligibility criteria and were therefore excluded. The remaining 90 participants and/or their parents/legal guardians received an invitation for further participation via email, and 33 accepted. The flow of participants through the study is summarized in Figure 2. Of those who accepted the invitation, 27 (26.2 ± 11.4 years) showed up for the first out of two laboratory visits. All returned to the second visit for measurements on resting metabolic rate (RMR) but seven did not follow instructions of arriving in a fasted state and therefore their RMR could only be estimated. Finally, 24 started the food and training registrations in the app and 20 had six or seven complete days registered. One was excluded from the analyses due to physiological effects of age (>50 years). Of the remaining 19 (26.5 ± 10.3 years), 15 had a valid RMR measurement. While majority of those who responded to the online questionnaire were ball sport athletes, they became a minority among those who completed all parts of the study. Moreover, no athlete from technical sports continued to the measurement phase.

### 3.2 Energy availability and dietary intakes

**FIGURE 3:**
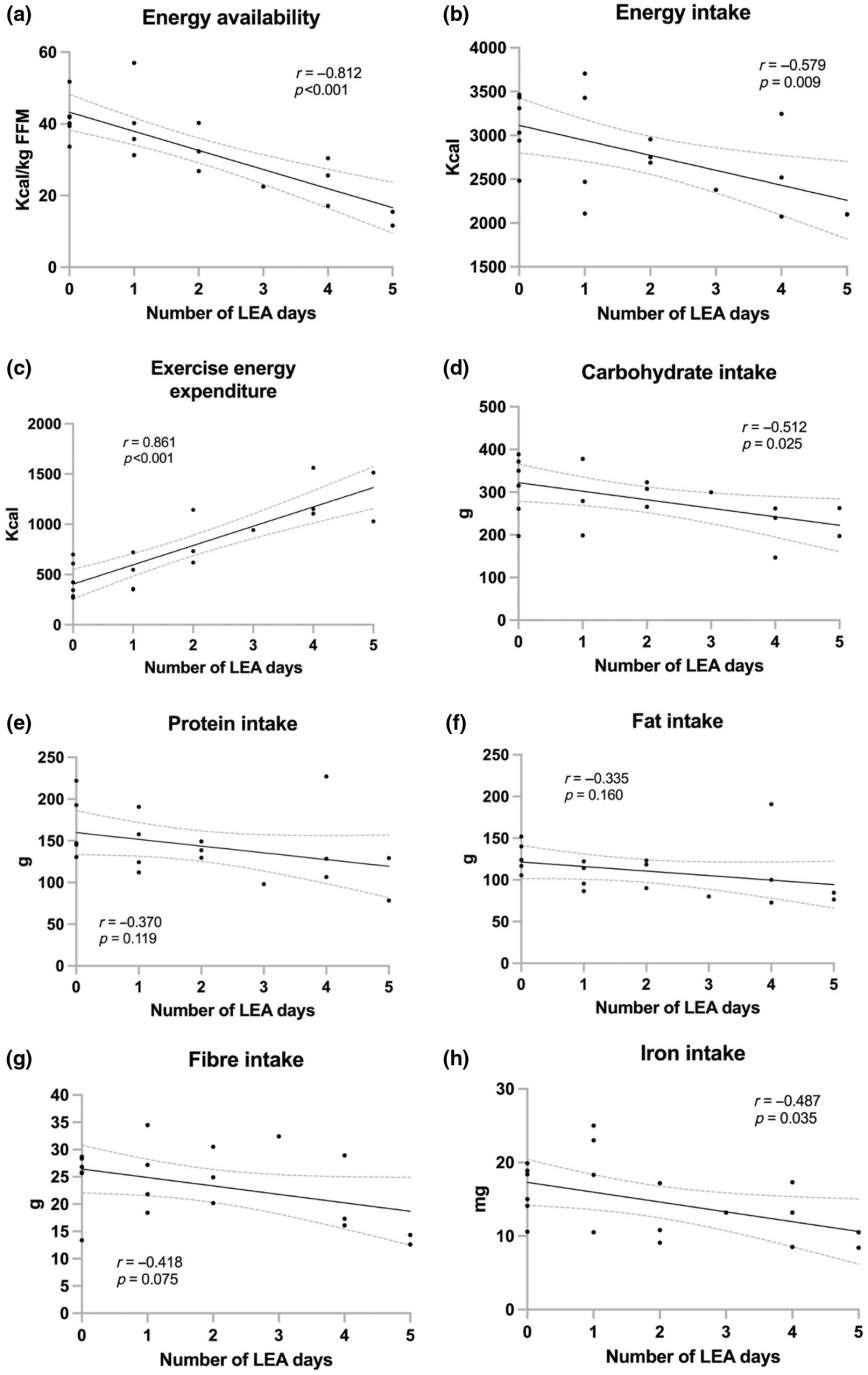
Correlations between number of low energy availability (LEA; EA <25 kcal/kg fat free mass) days with average (a) energy availability, (b) energy intake, (c) exercise energy expenditure, (d) carbohydrate intake, (e) protein intake, (f) fat intake, (g) fiber intake, and (h) iron intake. Correlations presented as Pearson coefficients (r) with best‐fit lines and 95% confidence bands.

For the 19 eligible athletes with 6–7 days registered in the app, the number of LEA days (EA <25 kcal/kg FFM) ranged between 0 and 5; 0 days (*n* = 6), 1 day (*n* = 4), 2 days (*n* = 3), 3 days (*n* = 1), 4 days (*n* = 3), and 5 days (*n* = 2). Average EA and relative macronutrient intakes are summarized in Table 1 and individual plots showing day‐to‐day patterns of EA and macronutrient intakes are shown in the File S1. Typical number of weekly training hours, derived from questionnaire, were 13.0 ± 5.0 (range: 7.5–26), while average training hours registered in the app were 11.1 ± 6.0 (range: 4–25). Number of LEA days were inversely correlated with EA, with lowest mean EA observed in those with 4–5 days of LEA (Figure 3a). In contrast, EEE was positively correlated with the number of LEA days, with mean EEE >1000 kcal/day for all athletes with 4–5 days of LEA (Figure 3c) while their energy intakes ranged from ~2100 to 3250 kcal/day (Figure 3b). Absolute carbohydrate intake was also inversely correlated with the number of LEA days (Figure 3d) but became non‐significant after adjusting for total bodyweight (*r* = −0.292, *p* = 0.224). Of evaluated micronutrient intakes, a significant inverse correlation was found between iron intakes and number of LEA days (Figure 3h) but not the other nutrients.

**TABLE 1:** Mean energy availability and dietary intakes of the 19 participants with 6–7 days registered.

	Mean ± SD	Range	Reference values^a^	*n* (%) below reference
Energy availability (kcal/kg FFM)	33.4 ± 11.8	11.6–57.0	25.0	4 (21.1)
Exercise energy expenditure (kcal)	758 ± 402	269–1562	‐	‐
Energy intake (kcal)				
Kcal	2799 ± 531	2075–3705	‐	‐
Kcal from supplements	175 ± 144	0–502	‐	‐
Carbohydrate intake				
g	285 ± 70	147–389	‐	‐
g/kg	4.0 ± 1.1	1.9–6.6	4.0	11 (57.9)
g from sport foods/supplements	21.0 ± 24.1	0–91	‐	‐
Protein intake				
g	145 ± 39	78–227	‐	‐
g/kg	2.0 ± 0.5	1.3–3.3	1.5	4 (21.1)
g from sport foods/supplements	15.7 ± 16.7	0–55.0	‐	‐
Fat intake				
g	111 ± 29	73–191	‐	‐
g/kg	1.6 ± 0.5	1.0–2.5	‐	‐
g from sport foods/supplements	3.0 ± 2.7	0–10.5	‐	‐
Fiber intake				
g	23.6 ± 6.7	12.6–34.5	25.0	8 (42.1)
g/kg	0.3 ± 0.1	0.2–0.5	‐	‐
g from sport foods/supplements	0.7 ± 1.0	0–2.9	‐	‐
Vitamin D intake (μg)				
Total	12.8 ± 9.2	3.4–35.7	15.0	14 (73.7)
From supplements	6.4 ± 8.7	0–27.8	‐	‐
Iron intake (mg)	14.8 ± 4.9	8.4–25.0	9.0	2 (10.5)
Folate intake (μg)	338 ± 99	170–653	300	5 (26.3)
Vitamin B12 intake (μg)	6.5 ± 3.5	2.7–18.9	3.2	1 (5.3)

*Note*: Data presented as means with standard deviations (SD) and value ranges (min‐max). Reference values and percentage of participants below the reference are also provided when applicable.

^a^ The lower end of athlete specific recommendations for energy availability, carbohydrate, and protein intake in male athletes. Reference values for fiber, vitamin D, iron, folate, and vitamin B12 are derived from the Icelandic and Nordic nutrition recommendations.

Vitamin D supplements were used by nine and their total vitamin D intakes averaged at 19.9 ± 8.8 μg compared to 6.3 ± 2.1 μg in those 10 that did not take vitamin D supplements (*p* < 0.001). Five used vitamin supplements without vitamin D, six minerals and/or electrolytes, and two used supplements with iron. Most (*n* = 15) reported using one or more type of supplemental protein; protein enriched dairy drinks (*n* = 10), protein powder (*n* = 7), and/or protein snacks (*n* = 8). Moreover, five reported using caffeine‐rich energy or pre‐workout drinks, five ergogenic aids (e.g., creatine), and seven exogeneous carbohydrates (e.g., gels or powders). Visual inspection did not reveal any trends in supplement patterns based on LEA days.

### 3.3 Body composition and physiological outcomes

**FIGURE 4:**
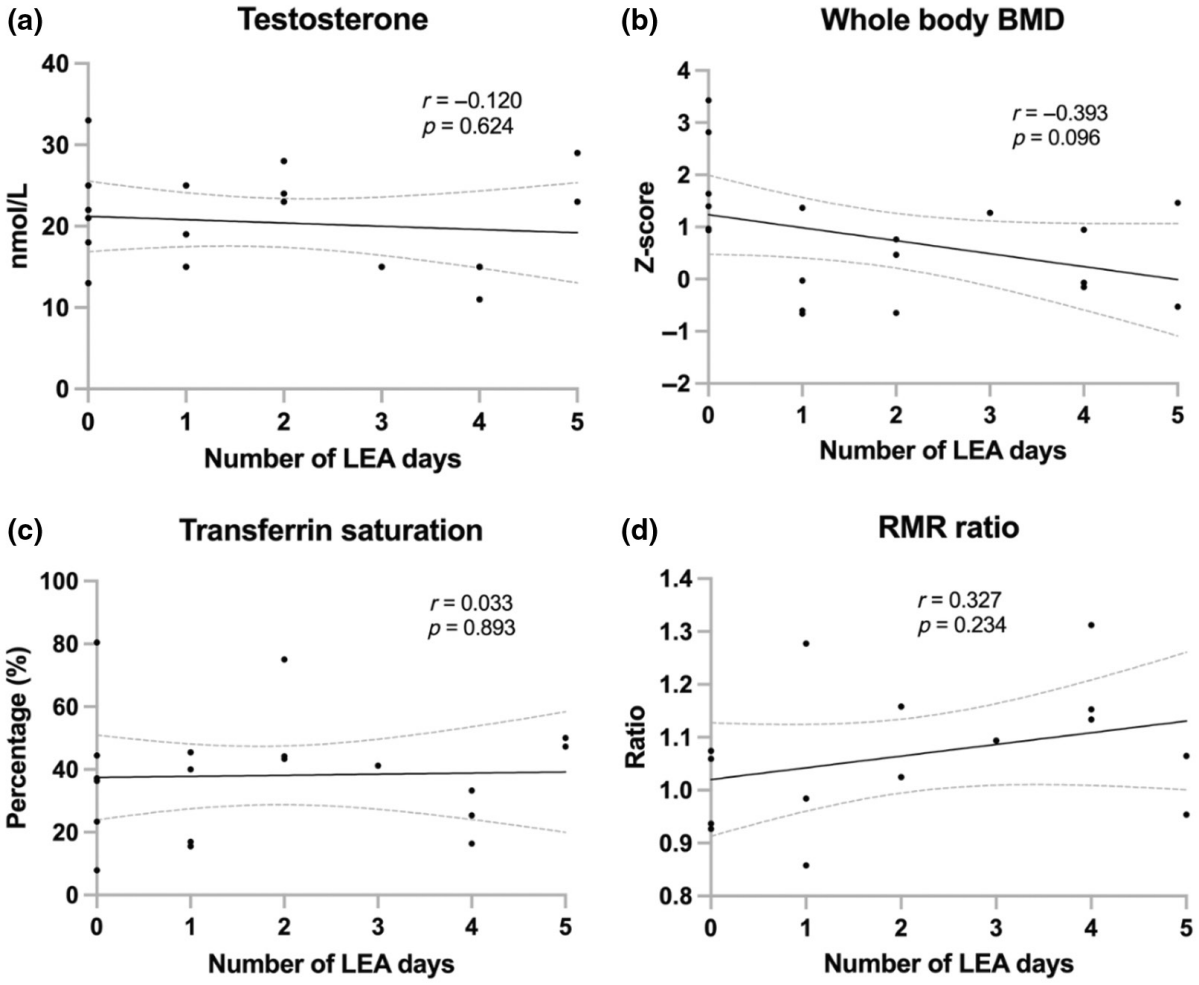
Correlations between number of low energy availability (LEA; EA <25 kcal/kg fat free mass) days with average (a) serum testosterone, (b) whole body bone mineral density (BMD), (c) transferrin saturation, and (d) resting metabolic rate (RMR) ratio. Correlations presented as Pearson coefficients (r) with best‐fit lines and 95% confidence bands.

Physiological characteristics and nutrition status of participants are presented in Table 2. None of the body composition nor any of the other physiological measures were associated with the number of LEA days (Figure 4). Moreover, no participant had clinically low levels of testosterone but one, with four LEA days, had sub‐clinically low levels (11 nmol/L) (Figure 4a). That athlete also had serum iron below the reference value, and low TSAT but did otherwise not display any other objective REDs symptoms. Highest *Z*‐scores for whole body BMD were observed among athletes with 0 days of LEA, but no athlete had score <−1 (Figure 4b). Moreover, no athlete had low ferritin levels while three had low levels of Fe and four TSAT <20% (Figure 4c). RMR ratio was <0.90 for one athlete which had one LEA day but no trends for associations of RMR ratio with number of LEA days were detected (Figure 4d). Serum 25(OH)D concentrations were below what has been recommended for athletes (<80 nmol/L) in 10 out of the 19, but none had vitamin D deficiency or insufficiency. 25(OH)D did not differ between those who reported using vitamin D supplements and those who did not (72.3 ± 10.5 vs. 84.6 ± 18.1 nmol/L, *p* = 0.089). None had TSH below reference range but four marginally exceeded the upper reference value. No athlete had IgA, calcium, and magnesium levels outside reference ranges, but two exceeded the reference range for vitamin B12.

**TABLE 2:** Physiological characteristics and nutrition status of the 19 participants with 6–7 days of food and training registrations.

	Mean ± SD	Range
Weight (kg)	72.9 ± 11.8	55.4–101.4
BMI (kg/m^2^)	22.4 ± 2.6	18.4–29.6
DXA FFM (kg)	60.5 ± 8.9	45.0–75.6
FFMI (kg/m^2^)	18.6 ± 1.8	15.2–22.1
DXA Fat mass (kg)	9.6 ± 3.5	5.1–21.7
DXA Body fat %	12.9 ± 3.1	8.2–21.4
DXA Whole body BMD *Z*‐score	0.78 ± 1.14	−0.7 to 3.4
RMR (kcal)^a^	1928 ± 177	1556–2242
RMR ratio^a^, ^b^	1.07 ± 0.13	0.86–1.31
Testosterone (nmol/L)	20.5 ± 6.1	11.0–33.0
TSH (mU/L)	2.3 ± 1.3	0.8–4.6
IgA (g/L)	2.0 ± 0.8	0.7–3.5
Fe (μmol/L)	19.6 ± 8.6	4.0–39.0
Ferritin (μg/L)	115 ± 60	34–243
TIBC (μmol/L)	52.9 ± 6.4	41–67
Transferrin saturation (%)	38.1 ± 18.7	7.8–80.5
Vitamin 25(OH)D (nmol/L)	78.8 ± 15.9	58.0–112.0
Calcium (mmol/L)	2.4 ± 0.1	2.3–2.7
Magnesium (mmol/L)	0.9 ± 0.1	0.8–1.0
Vitamin B12 (pmol/L)	468 ± 168	196–899

*Note*: Data presented as means with standard deviations (SD) and value ranges (min‐max).

Abbreviations: BMD, bone mineral density; BMI, body mass index; DXA, dual energy x‐ray absorptiometry; Fe, iron; FFM, fat free mass; FFMI, FFM index; IgA, immunoglobulin A; TIBC, total‐iron‐binding‐capacity; TSH, thyroid stimulating hormone.

^a^ Resting metabolic rate (RMR) measured via indirect calorimetry (valid measure available for 15 participants).

^b^ Calculated RMR ratio: measured RMR/estimated via the Cunningham formula.

Occurrences of negative self‐reported symptoms, among all 90 athletes that responded to LEAM‐Q, are summarized in the File S2 (Table S1). Of the 19 athletes that participated in all parts of the study, five had a low sex drive according to LEAM‐Q (two with 0 and the remaining three with 1, 2, and 4 LEA days) with all but one <18 years old. The one athlete with sub‐clinically low testosterone rated his sex drive as high.

### 3.4 Body image

**FIGURE 5:**
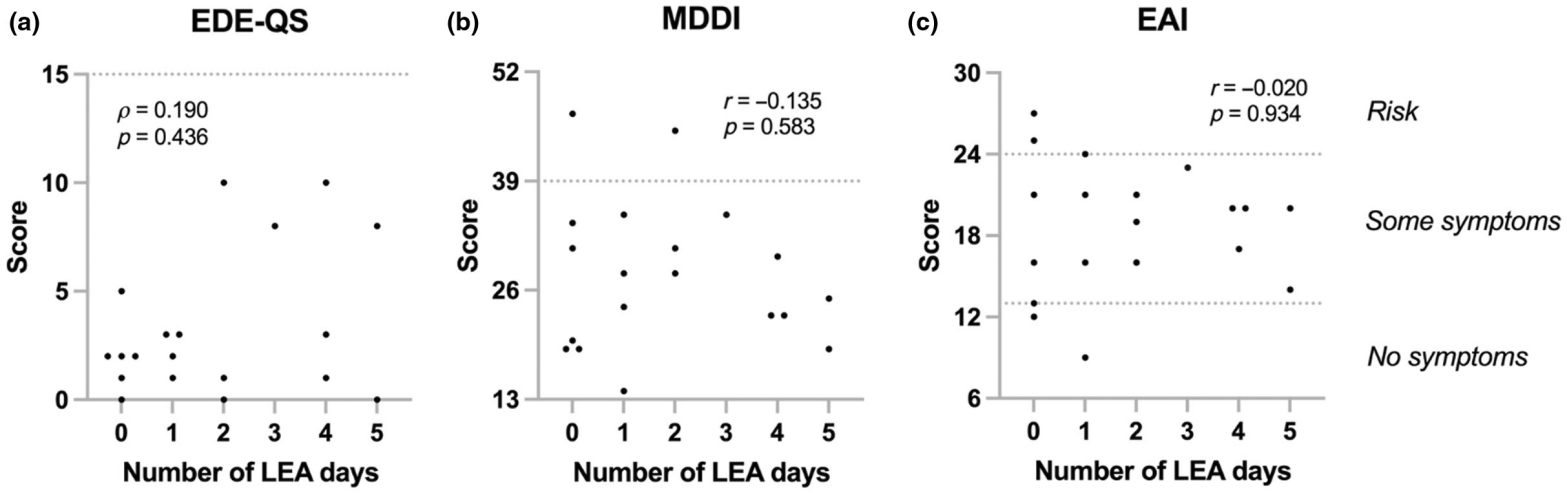
Correlations between number of low energy availability (LEA; EA <25 kcal/kg fat free mass) days and (a) Eating Disorder Examination‐Questionnaire Short (EDE‐QS), (b) Muscle Dysmorphic Disorder Inventory (MDDI), and (c) Exercise Addiction Inventory (EAI). Dotted lines represent the questionnaires' cutoffs. Correlations presented as Spearman's rank coefficients for EDE‐QS and Pearson coefficients (r) for MDDI and EAI.

The median EDE‐QS score was 2.0 (IQR: 1.0–5.0), with none exceeding the cutoff. However, those displaying most DE symptoms had ≥2 LEA days (Figure 5a). In accordance, the highest EDE‐QS score was 10, displayed by one aesthetic (four LEA days) and one ball sport (two LEA days) athlete. The mean MDDI score was 27.9 ± 8.8 (range: 14–47) and two exceeded the cutoff. The mean EAI score was 18.6 ± 4.7 (range: 9–27) and three were considered at risk, thereof one of those exceeding the MDDI cutoff. No correlations were found between the number of LEA days and scores on EDE‐QS, EAI, and MDDI (Figure 5a–c).

## 4 DISCUSSION

This study aimed to assess occurrences of LEA exposures in males from various sports over 7 days. Subsequently, associations of the number of LEA days with dietary intake, physiological measures, and body image concerns were evaluated. The number of LEA days were associated with higher EEE, and inversely with intakes of total energy, carbohydrates, and iron. Neither intakes of other nutrients, nor any of the physiological and body image measures were associated with the number of LEA days.

### 4.1 Energy availability and physiological outcomes

The weekly mean EA averaged at 33 kcal/kg FFM/day with four having mean values below 25, and additional two or in total around 30% below 30 kcal/kg FFM/day which is similar to earlier investigations assessing 3–7 days of EA in male populations (Jurov et al., 2021; Lane et al., 2019, 2021). Previous cross‐sectional investigations have reported no to inconclusive associations between mean EA and proposed biomarkers of REDs (Jurov et al., 2021; Lane et al., 2021). None of the athletes in this study had continuously low EA throughout the registered week (i.e., all 6–7 days). However, males with highest EEE appeared to have greatest number of LEA days. For example, there were cases of participants with mean daily EEE of up to 1500 kcal while their registered energy intakes marginally exceeded 2000 kcal on average, consequently resulting in low calculated EA. This agrees with a recent study describing vast day‐to‐day EA fluctuations in male cyclists, where high EEE were often insufficiently compensated by energy intakes (Taylor et al., 2022). It is likely that such mismatches, if frequently repeated over time, would become problematic. What remains to be defined is indeed the duration and degree of LEA resulting in disrupted physiological function and sport performance. Accordingly, only a few days of LEA exposures have been found to impact health and performance in females (Melin et al., 2023; Oxfeldt et al., 2023; Papageorgiou et al., 2017). The absent associations of the number of LEA days and the objective outcomes of interests indicate that REDs occurs after longer and/or more drastic LEA exposures in males versus females. In accordance, intervention studies on males have reported that physiological complications (i.e., problematic LEA) are primarily observed at the more extreme ends (Jurov, Keay, & Rauter, 2022; Sim et al., 2024). A recent study on 12 trained to well‐trained male endurance athletes used a three‐stage intervention with 14 days at each stage (EA reduced by 25%, 50%, and 75% from baseline) and one‐month wash‐out periods. They concluded that there was no evident cutoff but rather an EA range between 9 and 25 kcal/kg/FFM/day where detrimental effects occurred in their sample. Another interesting finding of that study was also that the athletes' wellbeing and performance were impacted before hormone concentrations decreased (Jurov, Keay, Spudić, & Rauter, 2022). Thus, the use of subjective measures may aid early detection of REDs. That partly contradicts with the finding that generated scores, other than those related to sex‐drive, for the newly developed LEAM‐Q are currently considered ineffective in identifying REDs cases (Lundy et al., 2022). Here we found no associations between the sex drive score and number of LEA days, perhaps because that score was validated in males ≥18 years of age while most of those defined as having low sex drive in the current study were <18 years old. As summarized in the supplement to this paper undesirable symptoms related to perceived wellbeing were prevalent in the larger RED‐I study. Future studies, accounting for age and sport‐specific factors may aid further development of valid screening tools for males (Lundy et al., 2022). Moreover, as the IOC recently published an updated clinical assessment tool or REDs CAT2 (Mountjoy et al., 2023; Stellingwerff et al., 2023), the present study may be replicated using the newly defined primary and secondary indicators. To date, one study on female football players only has assessed the risk of REDs according to REDs CAT2 (Dasa et al., 2024).

### 4.2 Dietary intakes and nutrition status

Continuous or prolonged LEA is not only related to suboptimal total energy intakes but also increases likelihood of low macro‐ and micronutrient intakes (Jordan et al., 2020; Vardardottir et al., 2024). That trend was indeed seen for carbohydrate, fiber, and iron intakes in the current study, but chronically low intakes of those and other nutrients would undoubtedly pose a threat to both health and performance (Jordan et al., 2020; Logue et al., 2018). Low carbohydrate availability, especially, has been suggested to increase the risk of REDs (Fensham et al., 2022; Jagim et al., 2022; McKay et al., 2022; Vardardottir et al., 2024). Absolute but not relative carbohydrate intakes were significantly associated with the number of LEA days in this study. Moreover, as for total energy intakes the athletes had considerable between‐day fluctuations in carbohydrate intakes. From screening the dietary and training records it also appeared that some of the athletes with highest number of LEA days often used exogeneous carbohydrates to fuel long training sessions. Such compensatory behaviors or strategies could theoretically mitigate the risk of REDs (Podlogar & Wallis, 2022).

There is limited to absent evidence for direct causal relationships between micronutrient status and REDs, although intakes from foods and dietary supplements are among contributors to blood concentrations of vitamin D, iron, and other micronutrients. Thus, sufficient nutrition status may be regarded as a protective factor while insufficiencies are likely to exacerbate the consequences of REDs (Jordan et al., 2020). Apart from three males having low serum iron and four low TSAT, no evident nutrient deficiencies were observed. The prevalence of iron deficiency has been estimated to be 5%–11% in male athletes (Sim et al., 2019). The athlete with sub‐clinically low testosterone also presented low serum iron and TSAT, but we have previously reported associations between iron status and testosterone (Vardardottir et al., 2023). Importantly though, both testosterone and iron status are not only influenced by dietary intakes, but also exercise‐related factors and adaptive mechanisms (Hackney, 2020; Sim et al., 2019). Vitamin D plays a major role for health and performance, while suboptimal status may further induce negative impact of REDs on bone health (Todd et al., 2015). A recent meta‐analysis of eight studies reported no significant sex differences in the prevalence of vitamin D insufficiency (<50 nmol/L) in elite athletes (male range: 0%–62%, female range: 0%–83%) (Harju et al., 2022). In contrast with results of our female study where approximately 20% had vitamin D insufficiency or deficiency no athlete in the present study was insufficient or deficient in vitamin D. However, half of them had levels below the athlete specific recommendation of 80 nmol/L but this percentage was 75% for the females (Vardardottir et al., 2024). The visual sex differences may partly be explained by the fact that only 29% (12 out of 41) in the female study supplemented vitamin D compared to a half of the males in this study. In addition, most male athletes were measured in the summer to early autumn, but most females came in the spring and early summer. Seasonal variation must therefore also be considered (Backx et al., 2017).

### 4.3 Body image concerns in males

Negative body image and DE behaviors are known risk factors of REDs (Mountjoy et al., 2023; Torstveit et al., 2019; Vardardottir et al., 2023). The reported incidence of ED is 0%–19% in male athletes and is generally found to be lower than in females (Oevreboe et al., 2023; Sundgot‐Borgen & Torstveit, 2004). However, much less focus has been placed on body image concerns and associated psychological traits in males. As addressed previously body image concerns are multifactorial but commonly used screening instruments are largely female centric (Vardardottir et al., 2023). A recent study on male and female Norwegian athletes reported a higher prevalence of obsessive‐compulsive disorder (OCD) or related disorders (18.9%; females 22.2%, males 15.4%) than ED (5.7%; females 11.1%, no male) based on a diagnostic interview. However, a slightly higher percentage or 8.5% exceeded the EDE‐QS cutoff. A total of ~14% and three out of four with OCD had a body dysmorphic disorder in that study (Oevreboe et al., 2023). That is in good agreement with our previous publication where we reported that 8.4% of the 83 participants (6/56 females, 1/27 males) who started the measurement phase of the RED‐I study exceeded the EDE‐QS and 13.3% the MDDI cutoff (eight females, three males). There we also reported significant associations of the MDDI scoring with calculated scores for fitness (assessing occurrence of symptoms such as bodily and muscle pains or stiffness, injury vulnerability, and physical exhaustion) and sleep on LEAM‐Q in the males (Vardardottir et al., 2023).

Although none of the athletes included in the present investigation exceeded the EDE‐QS cutoff a few reported symptoms such as strong desire to lose weight and/or that they feared gaining weight. All athletes with ≤1 LEA days scored low on EDE‐QS while highest scores were found for individual athletes with two to five LEA days. This suggests that body image concerns might to some extents have influenced dietary intakes and/or exercise behaviors, and consequently EA as well. The MDDI scores were more spread with one athlete with zero and one with two LEA days exceeding the cutoff. Some features are shared between restrictive ED and muscle dysmorphia, including leanness focus, fear of gaining body fat and adherence to excessive exercise and dietary behaviors. Therefore, one does not necessarily rule out the other (Lichtenstein et al., 2022; Vardardottir et al., 2023). Importantly, MDDI and similar screening tools for muscle dysmorphia have primarily been validated among bodybuilders and habitual exercisers but not specially in athletic populations (Cooper et al., 2020; Hildebrandt et al., 2004). More rigorous qualitative studies could aid understanding of how multifaceted body image concerns present in male athlete populations and how they may contribute to the development of REDs (Oevreboe et al., 2023; Vardardottir et al., 2023).

Finally, scoring on EAI was not associated with number of LEA days, but other studies have suggested that the link between compulsive exercise and REDs is mitigated by the absence of disordered or restrictive eating behaviors (Kuikman et al., 2021).

### 4.4 Contribution of practical challenges to the male specific knowledge gap

Males represent only around 20% of athletes included in REDs investigations worldwide, while their participation is dominant in other types of sport science research (Cowley et al., 2021; Mountjoy et al., 2023). The IOC recently urged scientific action to bridge the male‐specific knowledge gap, and increase awareness of REDs in male athletes (Hackney et al., 2023). Sex differences in athletes' interest and willingness to participate in research have been suggested, with females reported to be more interested in for example, psychological and mental health outcomes (Nuzzo & Deaner, 2023). The potentially more female appealing research questions of this project may partly explain why, despite the authors' intentions of reaching equal sex participation rates, one‐third% of those who participated in both parts of the study were males. (Vardardottir et al., 2023). Future concerns include how scientists can reach the relevant target group, especially males and ball sports athletes. It could also be argued that data driven athletes with high health literacy, and/or those who can afford the time and manage the logistics of a study are most likely to participate, resulting in a volunteer bias (Tripepi et al., 2010). Another potential influential factor in the present and similar studies is the interest, availability, and attitudes of parents/legal guardians and even coaches or other team members. Accordingly, the mean age increased from 23.5 to 26.5 years between the study parts, with 14 out of the 19 who had sufficient registrations in the app being older than 18 years. In contrast, adolescents (<18 years) represented approximately 36% of the 90 athletes with complete response to the online questionnaire.

### 4.5 Study limitations

A few limitations apply to this and other studies using a similar design. This includes that a week of EA assessments only provides a snapshot of individuals' lives and does not provide information on past LEA exposures nor between‐week variations. Another limitation is the relatively low number of athletes with sufficient dietary and training records to be included in the main analyses. It should also be mentioned that the athletes did not participate during predefined training seasons, but EEE commonly varies between seasons and this limitation could thus have interfered with the analyses. In addition, despite advantages of using photo‐supported mobile applications for dietary and training registrations, some errors are near inevitable, for example, due to the possibility of under‐ or overreporting. The reporting of max HR during training session was optional (as its measurement is not always allowed and/or practical during training and competitions) which may have made the EEE evaluation via assigned METs less accurate in athletes who did not report this. However, that limitation was partly compensated for by accounting for measured RMR. The athletes did not respond to LEAM‐Q at the same time they participated in the measurement phase, and thus it is possible that some would have responded differently to the sex‐drive items if the questionnaire had been administered at the time as the other data collection or repeated at that time. Other limitations of the greater RED‐I project have been thoroughly outlined elsewhere (Vardardottir et al., 2023, 2024).

## 5 CONCLUSION AND FUTURE DIRECTIONS

Considerable day‐to‐day EA fluctuations but not continuously low EA were observed in participants of this study. We did not find any associations between the number of LEA days with the physiological and body image outcomes, although those with greatest number of LEA days had highest EEE but relatively low dietary intakes. These findings must be confirmed by more robust studies, preferably including more male specific REDs markers or indicators. In a wider context, the evident male specific knowledge gap on REDs can only be adequately filled by considerable contribution from all stakeholders, including scientists, practitioners, and athletes themselves. Joint efforts can drive the advancement of our understanding on REDs in male athletes, fostering a healthier, more informed athletic community.

## AUTHOR CONTRIBUTIONS

Study design and conceptualization: BV, ASO, and SLG; Funding and ethical applications: BV, ASO, and SLG; Participant recruitment: BV; Conduct of experiments: BV; Data cleansing and analyses: BV; Data discussion and interpretation: BV, ASO, and SLG; Manuscript draft and graphical illustrations: BV; Manuscript editing: BV, ASO, and SLG. Study guarantor: SLG.

## FUNDING INFORMATION

The study received funding from the University of Iceland Research Fund, the University of Iceland Doctoral Grants Fund, Icelandic Sport Fund (222533‐2501), and the Icelandic Public Health Fund (P‐2020‐10‐13‐0042).

## CONFLICT OF INTEREST STATEMENT

None.

## ETHICS STATEMENT

The research protocol was approved by the Icelandic Ethics Committee (VSNb2021050006/03.01). All participants and parents/legal guardians of those under 18 years of age provided written informed consent prior to participation.

## Supporting information


File S1.

Table S1.

## Data Availability

All data relevant to the study are included in the article or uploaded as supplemental information.

## References

[phy216112-bib-0001] Ainsworth, B. E. , Haskell, W. L. , Whitt, M. C. , Irwin, M. L. , Swartz, A. M. , Strath, S. J. , Bassett, D. R., Jr. , Schmitz, K. H. , Emplaincourt, P. O. , Jacobs, D. R., Jr. , & Leon, A. S. (2000). Compendium of physical activities: An update of activity codes and MET intensities. Medicine and Science in Sports and Exercise, 32(9 Suppl), S498–S504. 10993420 10.1097/00005768-200009001-00009

[phy216112-bib-0002] Backx, E. , van der Avoort, C. , Tieland, M. , Maase, K. , Kies, A. , van Loon, L. , de Groot, L. , & Mensink, M. (2017). Seasonal variation in vitamin D status in elite athletes: A longitudinal study. International Journal of Sport Nutrition and Exercise Metabolism, 27(1), 6–10. 27710147 10.1123/ijsnem.2016-0177

[phy216112-bib-0003] Bjørke‐Monsen, A. L. , & Lysne, V. (2023). Vitamin B(12)–a scoping review for Nordic Nutrition Recommendations 2023. Food Nutrition Research, 67, 67. 10.29219/fnr.v67.10257 PMC10710864 38084149

[phy216112-bib-0004] Bjørke‐Monsen, A. L. , & Ueland, P. M. (2023). Folate–a scoping review for Nordic Nutrition Recommendations 2023. Food Nutrition Research, 67, 67. 10.29219/fnr.v67.10258 PMC10770645 38187793

[phy216112-bib-0005] Burke, L. M. , Ackerman, K. E. , Heikura, I. A. , Hackney, A. C. , & Stellingwerff, T. (2023). Mapping the complexities of relative energy deficiency in sport (REDs): Development of a physiological model by a subgroup of the International Olympic Committee (IOC) Consensus on REDs. British Journal of Sports Medicine, 57(17), 1098–1108. 37752007 10.1136/bjsports-2023-107335

[phy216112-bib-0068] Burke, L. M. , Hawley, J. A. , Wong, S. H. S. , & Jeukendrup, A. E. (2011). Carbohydrates for training and competition. Journal of Sports Sciences, 29(sup1), S17–S27. 10.1080/02640414.2011.585473. 21660838

[phy216112-bib-0006] Carlsen, H. , & Pajari, A. M. (2023). Dietary fiber–a scoping review for Nordic Nutrition Recommendations 2023. Food Nutrition Research, 67, 67. 10.29219/fnr.v67.9979 PMC10619389 37920675

[phy216112-bib-0007] Cooper, M. , Eddy, K. T. , Thomas, J. J. , Franko, D. L. , Carron‐Arthur, B. , Keshishian, A. C. , & Griffiths, K. M. (2020). Muscle dysmorphia: A systematic and meta‐analytic review of the literature to assess diagnostic validity. The International Journal of Eating Disorders, 53(10), 1583–1604. 32737999 10.1002/eat.23349

[phy216112-bib-0008] Cowley, E. S. , Olenick, A. A. , McNulty, K. L. , & Ross, E. Z. (2021). “Invisible sportswomen”: The sex data gap in sport and exercise science research. Women in Sport and Physical Activity Journal., 29(2), 146–151.

[phy216112-bib-0009] Cunningham, J. J. (1991). Body composition as a determinant of energy expenditure: A synthetic review and a proposed general prediction equation. The American Journal of Clinical Nutrition, 54(6), 963–969. 1957828 10.1093/ajcn/54.6.963

[phy216112-bib-0010] Dasa, M. S. , Friborg, O. , Kristoffersen, M. , Pettersen, G. , Sagen, J. V. , Torstveit, M. K. , Sundgot‐Borgen, J. , & Rosenvinge, J. H. (2024). Risk and prevalence of relative energy deficiency in sport (REDs) among professional female football players. European Journal of Sport Science, 1–16. https://www.frontiersin.org/journals/endocrinology/articles/10.3389/fendo.2020.00011/full 10.1002/ejsc.12129 PMC11235940 38956804

[phy216112-bib-0011] De Souza, M. J. , Koltun, K. J. , & Williams, N. I. (2019). The role of energy availability in reproductive function in the female athlete triad and extension of its effects to men: An initial working model of a similar syndrome in male athletes. Sports Medicine, 49(Suppl 2), 125–137. 31696452 10.1007/s40279-019-01217-3 PMC6901401

[phy216112-bib-0012] Domellöf, M. , & Sjöberg, A. (2024). Iron–a background article for the Nordic Nutrition Recommendations 2023. Food Nutrition Research, 68, 68. 10.29219/fnr.v68.10451 PMC10870973 38370116

[phy216112-bib-0013] Fensham, N. C. , Heikura, I. A. , McKay, A. K. A. , Tee, N. , Ackerman, K. E. , & Burke, L. M. (2022). Short‐term carbohydrate restriction impairs bone formation at rest and during prolonged exercise to a greater degree than low energy availability. Journal of Bone and Mineral Research, 37(10), 1915–1925. 35869933 10.1002/jbmr.4658 PMC9804216

[phy216112-bib-0014] Forrest, L. N. , Perkins, N. M. , Lavender, J. M. , & Smith, A. R. (2019). Using network analysis to identify central eating disorder symptoms among men. The International Journal of Eating Disorders, 52(8), 871–884. 31228298 10.1002/eat.23123

[phy216112-bib-0015] Fredericson, M. , Kussman, A. , Misra, M. , Barrack, M. T. , De Souza, M. J. , Kraus, E. , Koltun, K. J. , Williams, N. I. , Joy, E. , & Nattiv, A. (2021). The male athlete triad‐a consensus statement from the female and male athlete triad coalition part II: Diagnosis, treatment, and return‐to‐play. Clinical Journal of Sport Medicine, 31(4), 349–366. 34091538 10.1097/JSM.0000000000000948

[phy216112-bib-0016] Gideon, N. , Hawkes, N. , Mond, J. , Saunders, R. , Tchanturia, K. , & Serpell, L. (2016). Development and psychometric validation of the EDE‐QS, a 12 item short form of the eating disorder examination questionnaire (EDE‐Q). PLoS One, 11(5), e0152744. 27138364 10.1371/journal.pone.0152744 PMC4854480

[phy216112-bib-0017] Hackney, A. C. (2020). Hypogonadism in exercising males: Dysfunction or adaptive‐regulatory adjustment? Frontiers in Endocrinology, 11, 1–16. 32082255 10.3389/fendo.2020.00011 PMC7005256

[phy216112-bib-0018] Hackney, A. C. , Anna, K. M. , Kathryn, E. A. , Monica Klungland, T. , Louise, M. B. , & Margo, L. M. (2023). REDs alert: Male athletes be wary and scientists take action! British Journal of Sports Medicine, 57(17), 1066–1067. 37752009 10.1136/bjsports-2023-106719

[phy216112-bib-0019] Harju, T. , Gray, B. , Mavroedi, A. , Farooq, A. , & Reilly, J. J. (2022). Prevalence and novel risk factors for vitamin D insufficiency in elite athletes: Systematic review and meta‐analysis. European Journal of Nutrition, 61(8), 3857–3871. 35882673 10.1007/s00394-022-02967-z PMC9596536

[phy216112-bib-0020] Hildebrandt, T. , Langenbucher, J. , & Schlundt, D. G. (2004). Muscularity concerns among men: Development of attitudinal and perceptual measures. Body Image, 1(2), 169–181. 18089149 10.1016/j.bodyim.2004.01.001

[phy216112-bib-0021] Ihle, R. , & Loucks, A. B. (2004). Dose‐response relationships between energy availability and bone turnover in young exercising women. Journal of Bone and Mineral Research, 19(8), 1231–1240. 15231009 10.1359/JBMR.040410

[phy216112-bib-0022] Itkonen, S. T. , Andersen, R. , Björk, A. K. , Brugård Konde, Å. , Eneroth, H. , Erkkola, M. , Holvik, K. , Madar, A. A. , Meyer, H. E. , Tetens, I. , Torfadóttir, J. E. , Thórisdóttir, B. , & Lamberg‐Allardt, C. J. E. (2021). Vitamin D status and current policies to achieve adequate vitamin D intake in the Nordic countries. Scandinavian Journal of Public Health, 49(6), 616–627. 31916497 10.1177/1403494819896878

[phy216112-bib-0023] Jagim, A. R. , Fields, J. , Magee, M. K. , Kerksick, C. M. , & Jones, M. T. (2022). Contributing factors to low energy availability in female athletes: A narrative review of energy availability, training demands, nutrition barriers, body image, and disordered eating. Nutrients, 14(5), 986. 35267961 10.3390/nu14050986 PMC8912784

[phy216112-bib-0024] Jordan, S. L. , Albracht‐Schulte, K. , & Robert‐McComb, J. J. (2020). Micronutrient deficiency in athletes and inefficiency of supplementation: Is low energy availability a culprit? PharmaNutrition., 14, 100229.

[phy216112-bib-0025] Jurov, I. , Keay, N. , Hadžić, V. , Spudić, D. , & Rauter, S. (2021). Relationship between energy availability, energy conservation and cognitive restraint with performance measures in male endurance athletes. Journal of the International Society of Sports Nutrition, 18(1), 24. 33736663 10.1186/s12970-021-00419-3 PMC7977281

[phy216112-bib-0026] Jurov, I. , Keay, N. , & Rauter, S. (2022). Reducing energy availability in male endurance athletes: A randomized trial with a three‐step energy reduction. Journal of the International Society of Sports Nutrition, 19(1), 179–195. 35813848 10.1080/15502783.2022.2065111 PMC9261741

[phy216112-bib-0027] Jurov, I. , Keay, N. , Spudić, D. , & Rauter, S. (2022). Inducing low energy availability in trained endurance male athletes results in poorer explosive power. European Journal of Applied Physiology, 122(2), 503–513. 34825937 10.1007/s00421-021-04857-4 PMC8617370

[phy216112-bib-0028] Knuiman, P. , Hopman, M. T. E. , Verbruggen, C. , & Mensink, M. (2018). Protein and the adaptive response with endurance training: Wishful thinking or a competitive edge? Frontiers in Physiology, 9, 598. 29875696 10.3389/fphys.2018.00598 PMC5974122

[phy216112-bib-0029] Kuikman, M. A. , Mountjoy, M. , & Burr, J. F. (2021). Examining the relationship between exercise dependence, disordered eating, and low energy availability. Nutrients, 13(8), 1–12. https://www.mdpi.com/2072‐6643/13/8/2601 10.3390/nu13082601 PMC8398044 34444761

[phy216112-bib-0030] Kyle, U. G. , Schutz, Y. , Dupertuis, Y. M. , & Pichard, C. (2003). Body composition interpretation: Contributions of the fat‐free mass index and the body fat mass index. Nutrition, 19(7), 597–604. 12831945 10.1016/s0899-9007(03)00061-3

[phy216112-bib-0031] Lane, A. R. , Hackney, A. C. , Smith‐Ryan, A. , Kucera, K. , Registar‐Mihalik, J. , & Ondrak, K. (2019). Prevalence of low energy availability in competitively trained male endurance athletes. Medicina, 55(10), 665. 31581498 10.3390/medicina55100665 PMC6843850

[phy216112-bib-0032] Lane, A. R. , Hackney, A. C. , Smith‐Ryan, A. E. , Kucera, K. , Register‐Mihalik, J. K. , & Ondrak, K. (2021). Energy availability and RED‐S risk factors in competitive, non‐elite male endurance athletes. Transl Med Exerc Prescr., 1(1), 25–32. 34296227 PMC8294781

[phy216112-bib-0033] Lichtenstein, M. B. , Johansen, K. K. , Runge, E. , Hansen, M. B. , Holmberg, T. T. , & Tarp, K. (2022). Behind the athletic body: A clinical interview study of identification of eating disorder symptoms and diagnoses in elite athletes. BMJ Open Sport & Exercise Medicine, 8(2), e001265. 10.1136/bmjsem-2021-001265 PMC9214368 35813128

[phy216112-bib-0034] Logue, D. , Madigan, S. M. , Delahunt, E. , Heinen, M. , Mc Donnell, S. J. , & Corish, C. A. (2018). Low energy availability in athletes: A review of prevalence, dietary patterns, physiological health, and sports performance. Sports Medicine, 48(1), 73–96. 28983802 10.1007/s40279-017-0790-3

[phy216112-bib-0035] Loucks, A. B. , Kiens, B. , & Wright, H. H. (2011). Energy availability in athletes. Journal of Sports Sciences, 29(Suppl 1), S7–S15. 21793767 10.1080/02640414.2011.588958

[phy216112-bib-0036] Loucks, A. B. , & Thuma, J. R. (2003). Luteinizing hormone pulsatility is disrupted at a threshold of energy availability in regularly menstruating women. The Journal of Clinical Endocrinology and Metabolism, 88(1), 297–311. 12519869 10.1210/jc.2002-020369

[phy216112-bib-0037] Lundy, B. , Torstveit, M. K. , Stenqvist, T. B. , Burke, L. M. , Garthe, I. , Slater, G. J. , Ritz, C. , & Melin, A. K. (2022). Screening for low energy availability in male athletes: Attempted validation of LEAM‐Q. Nutrients, 14(9), 1–19. https://www.mdpi.com/2072‐6643/14/9/1873 10.3390/nu14091873 PMC9101736 35565840

[phy216112-bib-0038] Mathisen, T. F. , Ackland, T. , Burke, L. M. , Constantini, N. , Haudum, J. , Macnaughton, L. S. , Meyer, N. L. , Mountjoy, M. , Slater, G. , & Sundgot‐Borgen, J. (2023). Best practice recommendations for body composition considerations in sport to reduce health and performance risks: A critical review, original survey and expert opinion by a subgroup of the IOC consensus on relative energy deficiency in sport (REDs). British Journal of Sports Medicine, 57(17), 1148–1158. 37752006 10.1136/bjsports-2023-106812

[phy216112-bib-0039] McKay, A. K. A. , Peeling, P. , Pyne, D. B. , Tee, N. , Whitfield, J. , Sharma, A. P. , Heikura, I. A. , & Burke, L. M. (2022). Six days of low carbohydrate, not energy availability, alters the iron and immune response to exercise in elite athletes. Medicine and Science in Sports and Exercise, 54(3), 377–387. 34690285 10.1249/MSS.0000000000002819

[phy216112-bib-0040] Melin, A. , Tornberg, A. B. , Skouby, S. , Faber, J. , Ritz, C. , Sjödin, A. , & Sundgot‐Borgen, J. (2014). The LEAF questionnaire: A screening tool for the identification of female athletes at risk for the female athlete triad. British Journal of Sports Medicine, 48(7), 540–545. 24563388 10.1136/bjsports-2013-093240

[phy216112-bib-0041] Melin, A. K. , Areta, J. L. , Heikura, I. A. , Stellingwerff, T. , Torstveit, M. K. , & Hackney, A. C. (2023). Direct and indirect impact of low energy availability on sports performance. Scandinavian Journal of Medicine & Science in Sports, 34, 1–23. https://onlinelibrary.wiley.com/doi/10.1111/sms.14327 10.1111/sms.14327 36894187

[phy216112-bib-0042] Mountjoy, M. , Ackerman, K. E. , Bailey, D. M. , Burke, L. M. , Constantini, N. , Hackney, A. C. , Heikura, I. A. , Melin, A. , Pensgaard, A. M. , Stellingwerff, T. , Sundgot‐Borgen, J. K. , Torstveit, M. K. , Jacobsen, A. U. , Verhagen, E. , Budgett, R. , Engebretsen, L. , & Erdener, U. (2023). 2023 International Olympic Committee's (IOC) consensus statement on relative energy deficiency in sport (REDs). British Journal of Sports Medicine, 57(17), 1073–1097. 37752011 10.1136/bjsports-2023-106994

[phy216112-bib-0043] Mountjoy, M. , Sundgot‐Borgen, J. K. , Burke, L. M. , Ackerman, K. E. , Blauwet, C. , Constantini, N. , Lebrun, C. , Lundy, B. , Melin, A. K. , Meyer, N. L. , Sherman, R. T. , Tenforde, A. S. , Klungland Torstveit, M. , & Budgett, R. (2018). IOC consensus statement on relative energy deficiency in sport (RED‐S): 2018 update. British Journal of Sports Medicine, 52(11), 687–697. 29773536 10.1136/bjsports-2018-099193

[phy216112-bib-0044] Mujika, I. , Halson, S. , Burke, L. M. , Balagué, G. , & Farrow, D. (2018). An integrated, multifactorial approach to periodization for optimal performance in individual and team sports. International Journal of Sports Physiology and Performance, 13(5), 538–561. 29848161 10.1123/ijspp.2018-0093

[phy216112-bib-0045] Nuzzo, J. L. , & Deaner, R. O. (2023). Men and women differ in their interest and willingness to participate in exercise and sports science research. Scandinavian Journal of Medicine & Science in Sports, 33(9), 1850–1865. 37218686 10.1111/sms.14404

[phy216112-bib-0046] Oevreboe, T. H. , Ivarsson, A. , Sundgot‐Borgen, J. , Knudsen, A. K. S. , Reneflot, A. , & Pensgaard, A. M. (2023). Mental health problems in elite sport: The difference in the distribution of mental distress and mental disorders among a sample of Norwegian elite athletes. BMJ Open Sport & Exercise Medicine, 9(3), e001538. 10.1136/bmjsem-2023-001538 PMC10357714 37485002

[phy216112-bib-0047] Olafsdottir, A. S. , Hörnell, A. , Hedelin, M. , Waling, M. , Gunnarsdottir, I. , & Olsson, C. (2016). Development and validation of a photographic method to use for dietary assessment in school settings. PLoS One, 11(10), e0163970. 27711120 10.1371/journal.pone.0163970 PMC5053534

[phy216112-bib-0048] Oxfeldt, M. , Phillips, S. M. , Andersen, O. E. , Johansen, F. T. , Bangshaab, M. , Risikesan, J. , McKendry, J. , Melin, A. K. , & Hansen, M. (2023). Low energy availability reduces myofibrillar and sarcoplasmic muscle protein synthesis in trained females. The Journal of Physiology., 601, 3481–3497. 37329147 10.1113/JP284967

[phy216112-bib-0049] Papageorgiou, M. , Elliott‐Sale, K. J. , Parsons, A. , Tang, J. C. Y. , Greeves, J. P. , Fraser, W. D. , & Sale, C. (2017). Effects of reduced energy availability on bone metabolism in women and men. Bone, 105, 191–199. 28847532 10.1016/j.bone.2017.08.019

[phy216112-bib-0050] Podlogar, T. , & Wallis, G. A. (2022). New horizons in carbohydrate research and application for endurance athletes. Sports Medicine, 52(1), 5–23. 36173597 10.1007/s40279-022-01757-1 PMC9734239

[phy216112-bib-0051] Prnjak, K. , Jukic, I. , Mitchison, D. , Griffiths, S. , & Hay, P. (2022). Body image as a multidimensional concept: A systematic review of body image facets in eating disorders and muscle dysmorphia. Body Image, 42, 347–360. 35926364 10.1016/j.bodyim.2022.07.006

[phy216112-bib-0052] Reinke, S. , Taylor, W. R. , Duda, G. N. , von Haehling, S. , Reinke, P. , Volk, H.‐D. , Anker, S. D. , & Doehner, W. (2012). Absolute and functional iron deficiency in professional athletes during training and recovery. International Journal of Cardiology, 156(2), 186–191. 21145121 10.1016/j.ijcard.2010.10.139

[phy216112-bib-0053] Sim, A. D. , Tan, H. Q. , Ali, Y. , & Burns, S. F. (2024). Original investigation: Manipulating energy availability in male endurance runners: A randomised controlled trial. Applied Physiology, Nutrition, and Metabolism. Epub ahead of print. 10.1139/apnm-2024-0037 38713922

[phy216112-bib-0054] Sim, M. , Garvican‐Lewis, L. A. , Cox, G. R. , Govus, A. , McKay, A. K. A. , Stellingwerff, T. , & Peeling, P. (2019). Iron considerations for the athlete: A narrative review. European Journal of Applied Physiology, 119(7), 1463–1478. 31055680 10.1007/s00421-019-04157-y

[phy216112-bib-0055] Slater, G. , & Phillips, S. M. (2013). Nutrition guidelines for strength sports: Sprinting, weightlifting, throwing events, and bodybuilding (pp. 67–77). Routledge. 10.1080/02640414.2011.574722 21660839

[phy216112-bib-0056] Stellingwerff, T. , Mountjoy, M. , McCluskey, W. T. , Ackerman, K. E. , Verhagen, E. , & Heikura, I. A. (2023). Review of the scientific rationale, development and validation of the International Olympic Committee Relative Energy Deficiency in Sport Clinical Assessment Tool: V.2 (IOC REDs CAT2)—By a subgroup of the IOC consensus on REDs. British Journal of Sports Medicine, 57(17), 1109–1118. 37752002 10.1136/bjsports-2023-106914

[phy216112-bib-0057] Sterringer, T. , & Larson‐Meyer, D. E. (2022). RMR ratio as a surrogate marker for low energy availability. Current Nutrition Reports, 11(2), 263–272. 35080753 10.1007/s13668-021-00385-x

[phy216112-bib-0058] Sundgot‐Borgen, J. , & Torstveit, M. K. (2004). Prevalence of eating disorders in elite athletes is higher than in the general population. Clinical Journal of Sport Medicine, 14(1), 25–32. 14712163 10.1097/00042752-200401000-00005

[phy216112-bib-0059] Taylor, H. L. , Garabello, G. , Pugh, J. , Morton, J. , Langan‐Evans, C. , Louis, J. , Borgersen, R. , & Areta, J. L. (2022). Patterns of energy availability of free‐living athletes display day‐to‐day variability that is not reflected in laboratory‐based protocols: Insights from elite male road cyclists. Journal of Sports Sciences, 40(16), 1849–1856. 36062921 10.1080/02640414.2022.2115676

[phy216112-bib-0060] Terry, A. , Szabo, A. , & Griffiths, M. (2004). The exercise addiction inventory: A new brief screening tool. Addiction Research and Theory, 12(5), 489–499.

[phy216112-bib-0061] Todd, J. J. , Pourshahidi, L. K. , McSorley, E. M. , Madigan, S. M. , & Magee, P. J. (2015). Vitamin D: Recent advances and implications for athletes. Sports Medicine, 45(2), 213–229. 25252613 10.1007/s40279-014-0266-7

[phy216112-bib-0062] Torstveit, M. K. , Fahrenholtz, I. L. , Lichtenstein, M. B. , Stenqvist, T. B. , & Melin, A. K. (2019). Exercise dependence, eating disorder symptoms and biomarkers of relative energy deficiency in sports (RED‐S) among male endurance athletes. BMJ Open Sport & Exercise Medicine, 5(1), e000439. 10.1136/bmjsem-2018-000439 PMC6350749 30792881

[phy216112-bib-0063] Torstveit, M. K. , & Sundgot‐Borgen, J. (2005). The female athlete triad: Are elite athletes at increased risk? Medicine and Science in Sports and Exercise, 37(2), 184–193. 15692312 10.1249/01.mss.0000152677.60545.3a

[phy216112-bib-0064] Tripepi, G. , Jager, K. J. , Dekker, F. W. , & Zoccali, C. (2010). Selection bias and information bias in clinical research. Nephron. Clinical Practice, 115(2), c94–c99. 20407272 10.1159/000312871

[phy216112-bib-0065] Tuma, C. , Schick, A. , Pommerening, N. , Braun, H. , & Thevis, M. (2023). Effects of an individualized vs. standardized vitamin D supplementation on the 25(OH)D level in athletes. Nutrients, 15(22), 4747. 38004144 10.3390/nu15224747 PMC10675819

[phy216112-bib-0066] Vardardottir, B. , Gudmundsdottir, S. L. , Tryggvadottir, E. A. , & Olafsdottir, A. S. (2024). Patterns of energy availability and carbohydrate intake differentiate between adaptable and problematic low energy availability in female athletes. Frontiers in Sports and Active Living, 6, 1390558. 38783864 10.3389/fspor.2024.1390558 PMC11111999

[phy216112-bib-0067] Vardardottir, B. , Olafsdottir, A. S. , & Gudmundsdottir, S. L. (2023). Body dissatisfaction, disordered eating and exercise behaviours: Associations with symptoms of REDs in male and female athletes. BMJ Open Sport & Exercise Medicine, 9(4), e001731. 10.1136/bmjsem-2023-001731 PMC10860738 38348179

