# Supplementary material for: A real‐life snapshot: Evaluating exposures to low energy availability in male athletes from various sports
Source: Physiol Rep. 2024 Jun 23;12(12):e16112. doi: 10.14814/phy2.16112 (PMC11194298; doi:10.14814/phy2.16112)
Supplement: Supplementary file 1 — File S1. [file PHY2-12-e16112-s002.pdf]

Supplemental file

Physiological reports 2024; ”A real-life snapshot: evaluating exposures to low energy availability in male athletes from various sports”; Birna Vardardottir (biva@hi.is), Anna S. Olafsdottir, Sigridur Lara Gudmundsdottir; University of Iceland, Faculty of Health Promotion, Sport & Leisure Studies, Reykjavik, Iceland.

The figures below show A) patterns of energy availability (EA), energy intake (EI), and exercise energy expenditure (EEE) in kcal/kg FFM), and B) relative macronutrient intake for all participants with 6-7 days registered in the study app (n=19). Figures are arranged by the number of days with EA <25 kcal/kg FFM but their numbers are otherwise random (and different from participant IDs). The registration days (numbers) are shown on the x-axis. Dotted line on the energy availability figures represents suggested LEA threshold, and dotted line on the macronutrient figures represent the cut-off used for low intakes of carbohydrates (4 g/kg BW). Information about sport group and age are provided below the figures.

0 LEA days

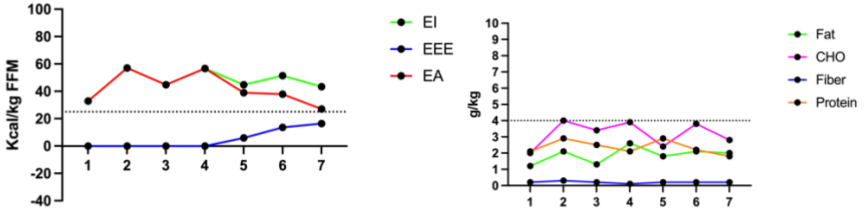

Athlete 1: power, 25 y.

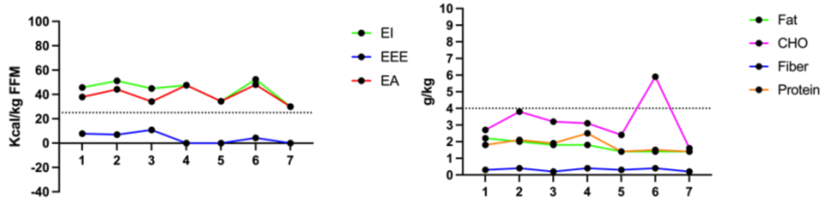

Athlete 2: endurance, 30 y.

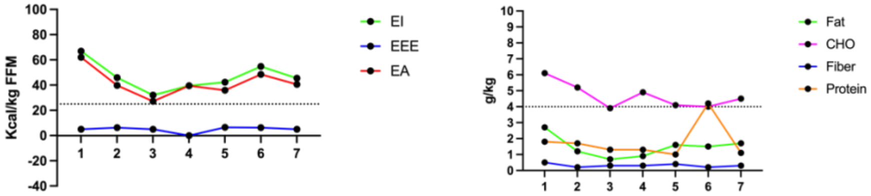

Athlete 3: power, 16 y.

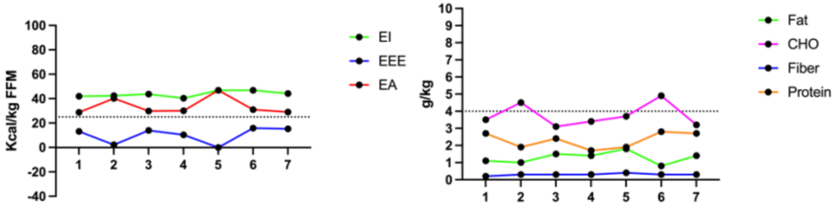

Athlete 4: ball, 23 y.

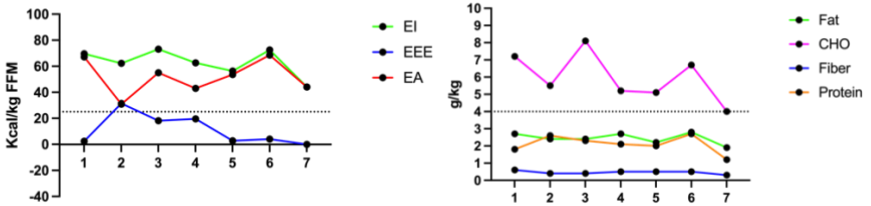

Athlete 5: endurance, 31 y.

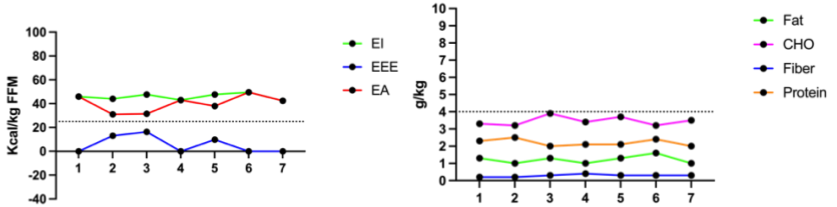

Athlete 6: weight-class, 49 y.

1 LEA day

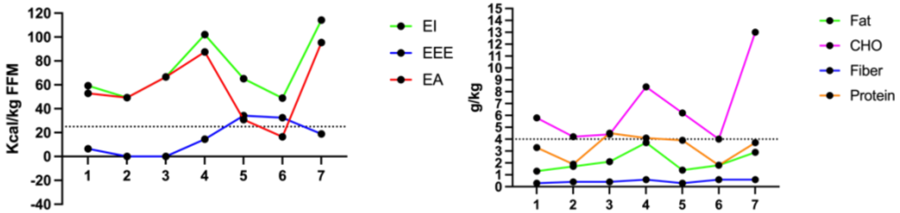

Athlete 7: endurance, 16 y.

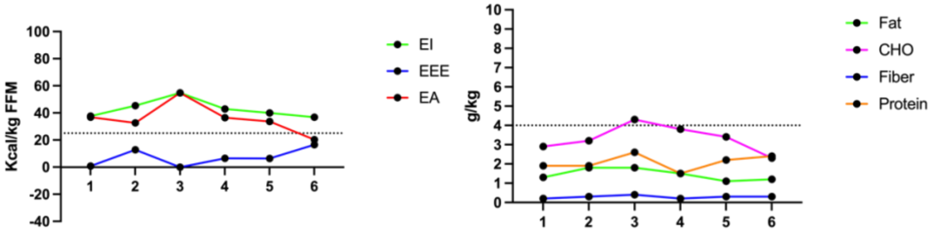

Athlete 8: endurance, 35 y.

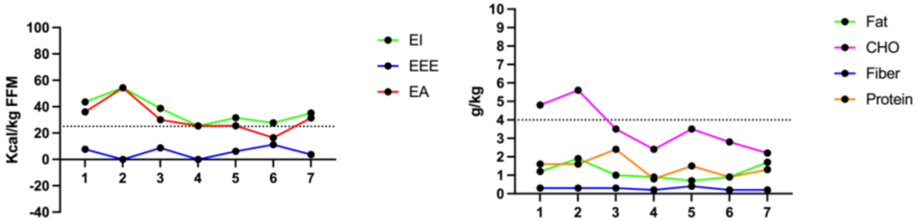

Athlete 9: power, 17 y.

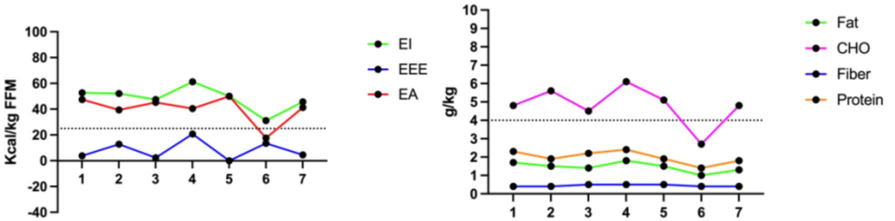

Athlete 10: endurance, 27 y.

2 LEA days

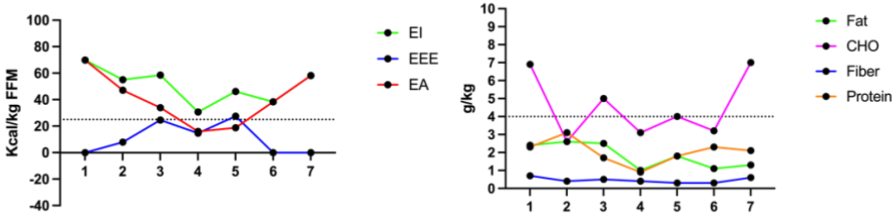

Athlete 11: aesthetic, 17 y.

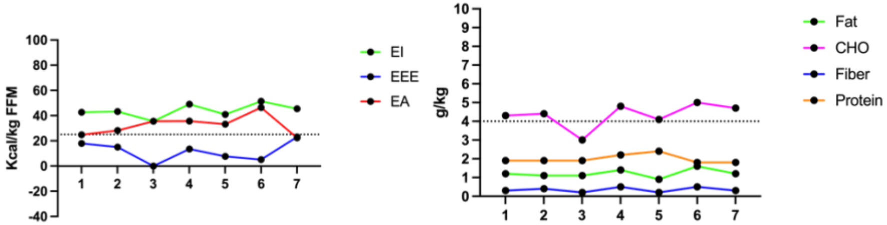

Athlete 12: ball, 27 y.

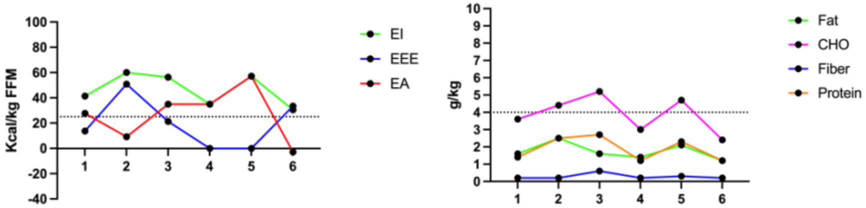

Athlete 13: weight-class, 18 y.

3 LEA days

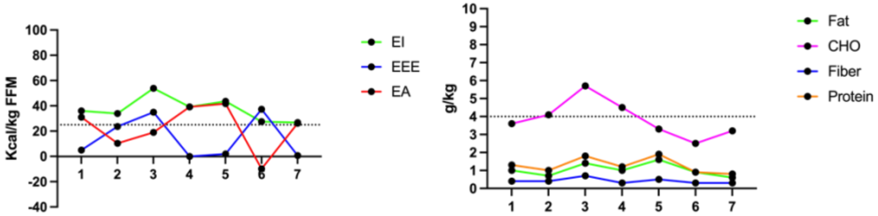

Athlete 14: Endurance, 50 y.

4 LEA days

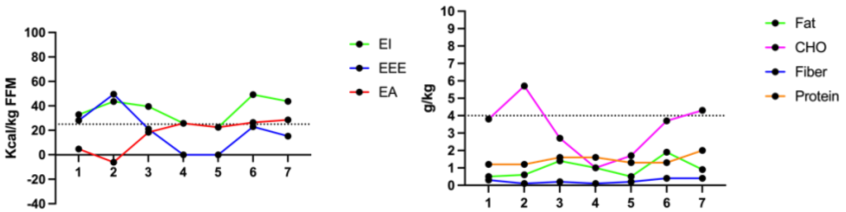

Athlete 15: endurance, 34 y.

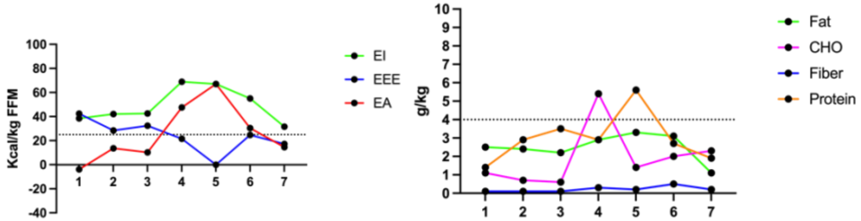

Athlete 16: Aesthetic, 19 y.

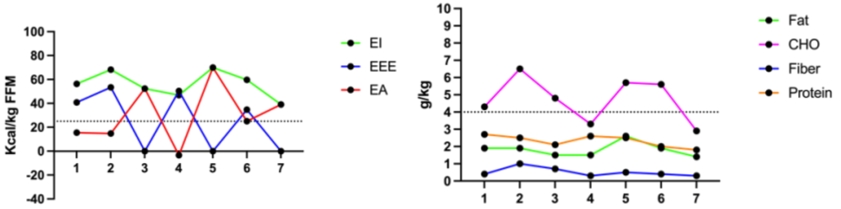

Athlete 17: Aesthetic, 15 y.

5 LEA days

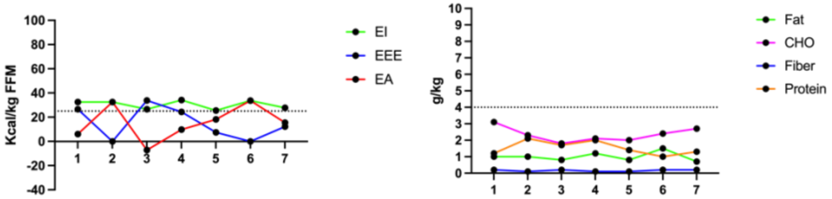

Athlete 18, power, 25 y.

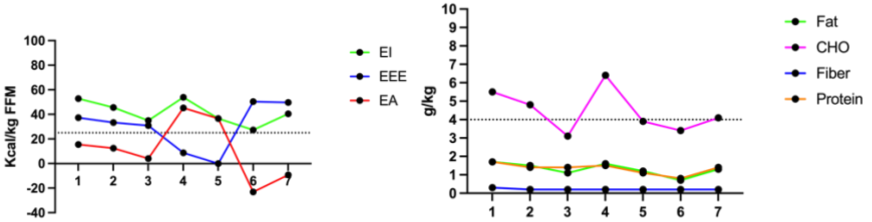

Athlete 19: aesthetic, 30 y.
