# Supplementary material for: A real‐life snapshot: Evaluating exposures to low energy availability in male athletes from various sports
Source: Physiol Rep. 2024 Jun 23;12(12):e16112. doi: 10.14814/phy2.16112 (PMC11194298; doi:10.14814/phy2.16112)
Supplement: Supplementary file 2 — Table S1. [file PHY2-12-e16112-s001.docx]

| **Table S1.** Prevalence of negative or undesirable symptoms based on responses to the Low Energy Availability in Males (LEAM-Q). Data presented as percentage of all males (n=90) in the RED-I study reporting that they often (several times a week to always) had a given negative symptom or rarely-to-never had a positive symptom. | | |
| --- | --- | --- |
|  |  | % |
| Dizziness | | |
| **Often** Feeling dizzy or ligtheaded when rising quickly |  | 7.8 |
| **Often** Problems with vision (e.g., blurring, seeing spots, tunnel vision) |  | 4.4 |
| Gastrointestinal symptoms | | |
| **Often** Feeling gaseous or bloated in the abdomen |  | 13.3 |
| **Often** Cramps or stomachache |  | 2.2 |
| Bowel movements every other day or less often |  | 6.7 |
| Normal stool: Diaorrhea like (watery) |  | 2.2 |
| Normal stool: Hard and dry |  | 1.1 |
| Thermoregulation at rest | | |
| **Often** Feeling very cold even when normally dressed |  | 6.7 |
| **Often** Dressed more warmly than companions regardless of weather |  | 11.1 |
| Fatigue | | |
| **Often** Feeling tired from work or school |  | 26.7 |
| **Often** Feeling overtired |  | 24.4 |
| **Often** Unable to concentrate well |  | 16.7 |
| **Often** Feeling lethargic |  | 7.8 |
| **Often** Put off making decisions |  | 21.1 |
| Fitness | | |
| **Often** Body parts are aching |  | 27.8 |
| **Often** Muscles feel stiff or tense during training |  | 14.4 |
| **Often** Muscle pain after performance |  | 6.7 |
| **Often** Feeling vulnerable to injuries |  | 10.0 |
| **Often** Having headache |  | 6.7 |
| **Often** Feeling physically exhausted |  | 14.4 |
| **Rarely/never** Feeling strong and making good progress with strength training |  | 21.1 |

| **Table S1.** continued. | | |
| --- | --- | --- |
|  |  | % |
| Sleep | | |
| **Rarely/never** Getting enough sleep |  | 17.8 |
| **Rarely/never** Falling asleep satisfied and relaxed |  | 14.4 |
| **Rarely/never** Waking up well rested |  | 26.7 |
| **Often** Sleeping restlessly |  | 17.8 |
| **Often** Sleep is easily interrupted |  | 17.8 |
| Average (absolute range) hours slept per night in the past month |  | 7.5 (4.5-9.5) |
| Recovery | | |
| **Rarely/never** Recovering well physically |  | 18.9 |
| **Rarely/never** Feeling in good physical shape |  | 6.7 |
| **Rarely/never** Achieving deserved progress in training and competition |  | 21.1 |
| **Rarely/never** Body feels strong |  | 14.4 |
| Energy levels | | |
| **Rarely/never** Feeling very energetic in general |  | 20.0 |
| **Rarely/never** Feeling invigorated for training sessions and  ready to perform well |  | 17.8 |
| **Rarely/never** Feeling happy and on top of life outside sport |  | 14.4 |
| **Often** Feeling down and less happy than usually or desired |  | 11.1 |
| Sex drive | | |
| General sex drive: low |  | 16.7 |
| General sex drive: not very interested in sex |  | 6.7 |
| Sex drive in the past month a little less than usual |  | 11.1 |
| Sex drive in the past month much less than usual |  | 5.6 |
| Morning erections 1-2 times per week in the past month |  | 36.7 |
| Morning erections rarely or never in the past month |  | 20.0 |
| Weekly morning erections in past month little less than usual |  | 10.0 |
| Weekly morning erections in past month much less than usual |  | 3.3 |

| **Table S1.** Continued. | |  |
| --- | --- | --- |
|  | % | |
| Number of acute injures in past 6 months |  | |
| None | 56.7 | |
| 1-2 | 35.6 | |
| 3-4 | 7.8 | |
| Number of overload injuries (same reoccuring overload injury counts as a new injury for every new period) in past 6 months |  | |
| None | 54.4 | |
| 1-2 | 31.1 | |
| 3-4 | 8.9 | |
| 5 or more | 5.6 | |
| Number of training breaks due to illness in past 6 months |  | |
| None | 46.7 | |
| 1-2 | 45.6 | |
| 3-4 | 3.3 | |
| 5 or more | 4.4 | |
| Max days in a row absent from training/competition or not able to perform optimally due to an injury or illness in past 6 months |  | |
| **Acute injury** |  | |
| None | 53.3 | |
| 1-7 days | 23.3 | |
| 8-14 days | 7.8 | |
| 15-21 days | 4.4 | |
| 22 days or more | 5.6 | |
| N/A | 5.6 | |
| **Overload injury** |  | |
| None | 51.1 | |
| 1-7 days | 28.9 | |
| 8-14 days | 2.2 | |
| 15-21 days | 6.7 | |
| 22 days or more | 3.3 | |
| N/A | 7.8 | |
| **Illness** |  | |
| None | 44.4 | |
| 1-7 days | 41.1 | |
| 8-14 days | 4.4 | |
| 15-21 days | 4.4 | |
| 22 days or more | 1.1 | |
| N/A | 4.4 | |
| Outcomes based on responses to the Low Energy Availability in Males Questionnaire (LEAM-Q). | |  |
